# Association of SGLT2 inhibitors use with a lower risk of biliary diseases in patients with type 2 diabetes mellitus: a retrospective cohort study

**DOI:** 10.1080/07853890.2026.2619263

**Published:** 2026-02-02

**Authors:** Ming Gao, Qiuyu Lin, Kaiyue Hu, Bei Zhong, Tingyi Zhu, Zheng Gong, Kaini Zhang, Xiaoli Chen, Xinyu Chen, Ying Zhang, Yangyang Li, Shaowen Tang, Dongming Su, Xiubin Liang, Yu Liu

**Affiliations:** ^a^ Department of Endocrinology, Sir Run Run Hospital, Nanjing Medical University, Nanjing, Jiangsu Province, China; ^b^ Department of Basic Medicine and Clinical Pharmacy, China Pharmaceutical University, Nanjing City, Jiangsu Province, China; ^c^ Department of Pathophysiology, Nanjing Medical University, Nanjing, Jiangsu Province, China; ^d^ Department of Ultrasound, Sir Run Run Hospital, Nanjing Medical University, Nanjing, Jiangsu Province, China; ^e^ Department of Epidemiology and Biostatistics, School of Public Health, Nanjing Medical University, Nanjing, Jiangsu Province, China; ^f^ Department of Pathology, Nanjing Medical University, Nanjing, Jiangsu Province, China

**Keywords:** Biliary diseases, cohort study, sodium-glucose cotransporter-2 inhibitors, type 2 diabetes

## Abstract

**Background:**

Type 2 diabetes mellitus (T2DM) increases the risk of biliary diseases (BD). This study aimed to evaluate the association between sodium-glucose cotransporter-2 inhibitors (SGLT2i) use andBD risk in T2DM patients, compared with sulfonylureas.

**Methods:**

We conducted a multi-center retrospective cohort study using electronic health records from Nanjing Medical University (January 2017-September 2022). Adults aged 18–75 years with T2DM newly prescribed SGLT2i or sulfonylureas were included. Propensity score with inverse probability of treatment weighting (IPTW) was applied to balance baseline covariates. Follow-up continued until BD onset, last visit, death, or study end. Cox proportional hazards models estimated hazard ratios (HRs) with 95% confidence intervals (CIs) complemented by sensitivity analyses, time-stratified analyses and subgroup analyses. Metabolic and biliary biomarkers were also compared.

**Results:**

A total of 1,901 T2DM patients were included (453 SGLT2i users and 1,448 sulfonylurea users). BD occurred in 24 SGLT2i users and in 188 sulfonylurea users. After IPTW adjustment, SGLT2i use was associated with a lower BD risk (HR 0.595, 95% CI 0.410–0.863, *p* = 0.020), remaining significant beyond 24 months and was stronger in patients aged over 60 years, without diabetic complications, or with high comorbidity burdens, consistent regardless of prior biguanides use. Glycemic control and body weight remained comparable, whereas SGLT2i users showed higher high-density lipoprotein cholesterol and lower total bile acid, and bilirubin levels.

**Conclusion:**

SGLT2i use was associated with a lower BD risk in T2DM patients, particularly with long-term treatment and among high-risk subgroups. Improved biliary biomarkers paralleled this reduction, suggesting a potential link that warrants further study.

## Introduction

Biliary diseases (BD) including gallstones (cholelithiasis), cholecystitis, cholangitis, biliary obstruction due to strictures or tumors, primary sclerosing cholangitis (PSC), primary biliary cholangitis (PBC), biliary cirrhosis, and biliary dyskinesia, represent a broad spectrum of hepatobiliary disorders that impose a substantial burden on global healthcare systems [1]. A recent meta-analysis, involving 115 studies and over 32 million participants estimated the global prevalence of gallstones at 6.1% (95% confidence intervals (CI), 5.6–6.5), with an incidence of 0.47 per 100 person-years, both of which increase with age [2]. Among known risk factors, diabetes mellitus has been consistently associated with a heightened risk of BD. Individuals with type 2 diabetes mellitus (T2DM) exhibit a significantly higher prevalence of gallstones (15.4%) compared to non-diabetic populations [3], potentially due to hyperinsulinemia, dyslipidemia, obesity and altered gallbladder motility. Despite the availability of various glucose-lowering therapies, the incidence of BD remains unacceptably high in individuals with T2DM. This highlights the unmet need for antidiabetic agents that not only control glycemia but also confer protection against biliary complications. Preventing BD in diabetic populations could reduce the need for surgical interventions, lower complication rates, and improve overall quality of life.

Interestingly, some glucose-lowering drugs such as insulin, dipeptidyl peptidase-4 inhibitors (DPP4i), and glucagon-like peptide-1 receptor agonists (GLP-1RA) have been associated with an increased risk of BD, including cholelithiasis [4–6]. These findings underscore the importance of identifying therapeutic strategies that target extra-glycemic pathways to reduce BD risk.

Sodium-glucose cotransporter-2 inhibitors (SGLT2i) represent a novel class of antidiabetic agents that lower blood glucose by enhancing urinary glucose excretion. Beyond glycemic control, SGLT2i have shown pleiotropic benefits in obesity, dyslipidemia, hyperuricemia, non-alcoholic fatty liver disease, osteoporosis, and cardiorenal protection [7–11], which may theoretically affect BD development. Intriguingly, SGLT2i have been associated with a reduced risk of kidney stones in T2DM patients, suggesting a broader influence on excretory and metabolic pathways [12]. However, their impact on BD has not been well characterized.

Recent mechanistic studies have suggested that SGLT2i may influence hepatic and bile acid metabolism. Empagliflozin has been shown to alleviate metabolic dysfunction–associated steatohepatitis (MASH) by promoting ketogenesis and suppressing CD8^+^ T cell-mediated inflammation [13], while dapagliflozin activates the AMPK-SIRT1 pathway, enhances autophagy, and reduces hepatic lipid accumulation [14]. These findings indicate that SGLT2i may improve bile acid homeostasis and liver lipid metabolism through multiple metabolic and inflammatory pathways, thereby providing a plausible biological basis for their potential protective effects against BD.

In light of these observations, we hypothesized that SGLT2i may reduce the risk of BD. This study aimed to evaluate whether SGLT2i use is associated with a reduced incidence of BD in patients with T2DM, compared to sulfonylureas, using real-world data and robust analytic methods. Our findings may reveal an unrecognized benefit of SGLT2i and provide new insights into its extra-glycemic effects.

## Methods

### Study design

We conducted a retrospective, population-based cohort study utilizing electronic health records from four hospitals affiliated with Nanjing Medical University (NMU): Sir Run Run Hospital, the Affiliated Eye Hospital, the Geriatric Hospital, and the Fourth Affiliated Hospital. The study covered the period from January 1, 2017, to September 1, 2022. The majority of cases were sourced from Sir Run Run Hospital of NMU, which also served as the coordinating center for the data collection. Patients who met the following criteria were included: (1) patients with T2DM; (2) patients newly treated with SGLT2i (including canagliflozin, empagliflozin, and dapagliflozin) or sulfonylureas (SU) (including glipizide, gliclazide, gliquidone, glimepiride, and glyburide) from January 1, 2017 to September 1, 2022; (3) patients with complete hospitalization information. Patients were excluded if they met any of the following criteria: (1) patients with age under 18 years or over 75 years; (2) pre-menopausal women, to minimize potential hormonal confounding, as estrogen fluctuations substantially influence biliary cholesterol saturation and gallstone formation; (3) patients with estimated glomerular filtration rate (eGFR) under 45 mL/min/1.73m^2^; (4) patients who have ever used DPP4i or GLP-1RA (which have already been reported to be associated with the occurrence of BD [4,15,16]); (5) patients with prevalent BD cases; (6) patients with uncertain diagnosis of BD during the study period; (7) patients with incomplete information during study; (8) patients who were lack of medical information before the index date; (9) patients who was treated with both SGLT2i and sulfonylureas; (10) patients who took less than one course of targeted medication; (11) patients who using other types of hypoglycemic drugs in the index date. All patient data were anonymized before analysis, and no identifiable information was retained.

The index date was defined as the first prescription of SGLT2i or sulfonylureas, with new users having no prior prescription for either drug in the preceding 6 months before this date. We selected sulfonylureas as the comparison group because sulfonylureas is the earliest and most widely used oral hypoglycemic agent in the treatment of diabetes, with the largest variety and established efficacy. It has been recommended as first-line alternative or second-line antidiabetic medication by many domestic and foreign guidelines for adult T2DM prevention and treatment and is often selected as a comparison group [17–19]. The primary outcome was defined as the first occurrence of biliary diseases, including cholecystitis, gallbladder polyps, and biliary stones, as diagnosed by International Classification of Diseases, Ninth Revision, Clinical Modification (ICD-9-CM) and Tenth Revision ICD-10-CM codes (Table S1). Diagnoses were primarily determined from electronic health records, and in a subset of patients, these codes were cross-validated with imaging reports, demonstrating high concordance. Secondary endpoints included (a) diagnosis of biliary diseases during follow-up; (b) switch from SGLT2i to sulfonylureas or vice versa, (c) SGLT2i enhancement with sulfonylureas or vice versa, (d) last clinical visit, (e) death record, and (f) end of the entire study (September 1, 2022).

The adapted Diabetes Complications and Severity Index (aDCSI) was used to evaluate the complications and severity of the diabetic patients [20]. The age-adjusted Charlson Comorbidity Index (aCCI) score, which is a widely used comorbidity scoring system, was included as a composite score to describe each patient’s disease burdens [21]. The specific scoring criteria are shown in Table S2. In addition, we included measurement of biochemistry, including fasting blood glucose (FBG), Glycosylated Hemoglobin (HbA1C), total cholesterol (TC), triglyceride (TG), low density lipoprotein cholesterol (LDLC), high density lipoprotein cholesterol (HDLC), total bile acid (TBA), total bilirubin (TBIL), direct bilirubin (DBIL), indirect bilirubin (IBIL), prior to the index date and data at termination of follow-up date. Details of the diabetic complications and antidiabetic medications are demonstrated in Tables S2 and S3.

### Statistical analyses

To mitigate the impact of confounding factors, we employed a propensity score with inverse probability of treatment weighting (IPTW) to create more comparable groups and reduce selection bias [22–24]. The propensity score (PS), derived from a logistic regression model encompassing demographics, comorbidities, complications, and medications, gauged the likelihood of each subject receiving SGLT2i treatment. Post PS calculation, individuals with extreme PS values beyond 1% thresholds were excluded to prevent undue influence on IPTW weights and enhance comparability between treatment groups [25]. Absolute standardized mean differences (ASMD) were computed to assess differences between comparison groups, with ASMD > 0.1 indicating notable distinctions [26]. The ASMD of all patients post 1% exclusion with IPTW is depicted in Figure S1. We determined the incidence density of biliary diseases per 1000 person-years. Cox proportional hazards models estimated hazard ratios (HRs) and robust 95% confidence intervals (CI) for risk of BD, and the proportional hazards assumption was tested using the stratified log-rank test with sulfonylurea consumption as the stratification factor [27]. The sulfonylureas treatment group served as the reference. Kaplan–Meier curves were employed for visualizing cumulative disease-free rates in both groups during follow-up. Besides, sensitivity analyses and subgroup analyses were conducted to verify the robustness of results. Firstly, alternative propensity scoring methods, including propensity-score matching (PSM) with caliper matching at 1:1, 1:2, 1:3 (caliper value 0.05), and propensity score with standardized mortality ratio weighting (SMRW) were employed to mitigate confounding effects [22,24]. Secondly, multivariable statistical methods such as logistic regression were applied to assess the relationship between treatment groups and biliary diseases risk. Thirdly, patients who developed biliary diseases within 6, 12, 18, or 24 months post-index date were excluded to mitigate misclassification bias. Additionally, subgroup analyses were conducted to provide more precise estimates of biliary diseases risk associated with SGLT2i, stratified by age (≤60 or >60 years old, a cutoff chosen according to the World Health Organization and the Law on the Protection of the Rights and Interests of the Elderly in China, which define individuals aged ≥60 years as older adults, to reflect the increasing biliary disease risk observed with advancing age [28]), presence of diabetic complications (any or none), clinical comorbidity status (low or high based on aCCI score, where low denotes aCCI < 5 points), type of SGLT2i (canagliflozin or non-canagliflozin), and prior use of biguanides (metformin). Because the concomitant use of insulin, thiazolidinediones (TZDs), alpha glucosidase inhibitor (AGIs), and other oral agents was already included in the propensity score model, additional subgroup analyses by these agents were not performed. A specific subgroup analysis was conducted for biguanides to address potential confounding related to its widespread use as first-line therapy. Results with a two-sided p value < 0.05 were deemed statistically significant. All statistical and sensitivity analyses were performed using R software (Version x64 4.1.3).

Additional analysis for sulfonylurea exposure. To assess whether the primary association could be driven by a potential increase in BD risk related to sulfonylureas, we performed an additional comparison between the sulfonylurea-treated group (SU) and an active-treated comparator cohort exposed to neither sulfonylureas nor SGLT2i. All patients included in this supplementary analysis were selected from the same source population and met the inclusion and exclusion criteria defined above for the T2DM cohort, using the same baseline assessment window and outcome ascertainment rules as the primary analysis. The SU group comprised patients treated with sulfonylureas with no exposure to SGLT2i before or after the index date. The ‘Neither SU nor SGLT2i’ group comprised patients receiving other glucose-lowering therapies but with no exposure to sulfonylureas or SGLT2i throughout follow-up. Baseline characteristics were summarized and compared between groups (Table S4). We calculated crude incidence density per 1,000 person-years and the corresponding incidence rate ratio (IRR). Time-to-event analyses were conducted using Cox proportional hazards models (unadjusted and adjusted for age, sex, aCCI, and baseline biguanides use). In addition, an adjusted logistic regression model using the same covariates was fitted as a complementary analysis (Table S5).

**Figure 1.**
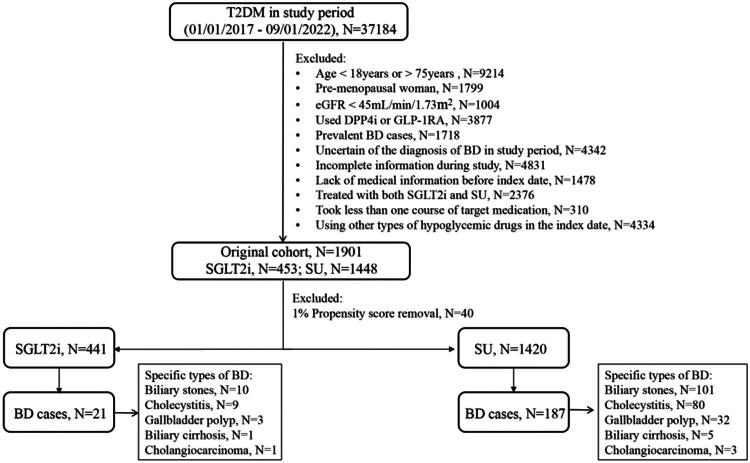
Flowchart of the study.

The flow chart is shown in Figure 1.

### Ethics approval and consent to participate

All research was conducted in accordance with the Declaration of Helsinki. This retrospective multicenter study was approved by the Institutional Review Board (IRB) of Sir Run Run Hospital, Nanjing medical university with the approval number 2024-SR-040. The IRB granted a waiver of informed consent due to the retrospective nature of the study and the use of fully anonymized data.

Ethical approvals from the other participating centers were not available; however, data were collected under institutional data-sharing agreements and were fully anonymized prior to transfer to the coordinating center. No direct patient contact occurred at any stage of the study.

All authors had access to the study data and had reviewed and approved the final manuscript.

## Results

### Characteristics of T2DM patients treated with SGLT2i or sulfonylureas

Our database included 37,184 T2DM patients from January 1, 2017, to September 1, 2022. Out of these, 1,901 individuals met the criteria for inclusion in our study, with 453 initiating SGLT2i and 1,448 initiating sulfonylureas (SU) as shown in Figure 1. This numerical difference reflects the real-world prescribing patterns during the study period, as sulfonylureas remained more commonly used than SGLT2i in routine clinical practice. Both groups were free of BD at baseline, with a mean follow-up of 2.83 years.

We compiled baseline characteristics of the participants, which are presented as counts and percentages, or as means with standard deviations (SD), in Table 1. The age of new users of SGLT2i had a mean (SD) of 59.64 (10.3) years and the aCCI score average (SD) was 5.67 (2.0). These statistics were comparable to those of new sulfonylurea users, who had a mean (SD) age of 62.04 (9.2) years and a mean (SD) aCCI score of 5.55 (1.8). In the SGLT2i group, 61.9% of patients had at least one diabetic complication, a percentage that was closely paralleled by the 55.4% in the sulfonylureas group.

**Table 1.** Baseline characteristics of patients treated with SGLT2 inhibitors or sulfonylureas before and after IPTW adjustment (1% trimming applied).

	Before IPTW with 1% removal				After IPTW with 1% removal			
	SGLT2i	SU	*p*	ASMD	SGLT2i	SU	*p*	ASMD
*N*	453 (23.8)	1448(76.2)	–	–	441(23.7)	1420(76.3)	–	–
Sex, male, *n* (%)	307(67.8)	920(63.5)	0.112	0.089	297 (67.3)	905 (63.7)	0.892	0.009
Age, mean (SD)	59.48(10.3)	61.95(9.4)	<0.001	0.251^a^	59.64(10.3)	62.04(9.2)	0.983	0.001
Smoke, *n* (%)	195(43.0)	569(39.3)	0.172	0.076	191(43.3)	560(39.4)	0.812	0.015
Drink, *n* (%)	143(31.6)	474(32.7)	0.685	0.025	139(31.5)	464(32.7)	0.867	0.011
**Diabetic co-medication, *n* (%)**								
Insulin	336(74.2)	637(44.0)	<0.001	0.645^a^	325(73.7)	622(43.8)	0.828	0.014
Biguanides	353(77.9)	1014(70.0)	0.001	0.181^a^	344(78.0)	994(70.0)	0.680	0.029
TZDs	36(7.9)	99(6.8)	0.485	0.042	36(8.2)	98(6.9)	0.698	0.024
AGI	199(43.9)	690(47.7)	0.183	0.075	196(44.4)	678(47.7)	0.549	0.039
NSUR	51(11.3)	82(5.7)	<0.001	0.202^a^	46(10.4)	79(5.6)	0.279	0.063
**Diabetic complications, *n* (%)**								
aDCSI group	284(62.7)	809(55.9)	0.012	0.139^a^	273(61.9)	787(55.4)	0.956	0.004
Diabetic retinopathy	50(11.0)	107(7.4)	0.018	0.126^a^	45(10.2)	104(7.3)	0.863	0.010
Diabetic neuropathy	43(9.5)	107(7.4)	0.177	0.076	39(8.8)	101(7.1)	0.931	0.005
Diabetic nephropathy	43(9.5)	100(6.9)	0.086	0.094	35(7.9)	93(6.5)	0.403	0.045
Stroke	228(50.3)	638(44.1)	0.022	0.126^a^	220(49.9)	619(43.6)	0.668	0.028
Metabolic emergency	14(3.1)	26(1.8)	0.137	0.084	12(2.7)	25(1.8)	0.745	0.018
Cardiovascular disease	1(0.2)	11(0.8)	0.355	0.077	1(0.2)	10(0.7)	0.156	0.072
OSAHS	9(2.0)	10(0.7)	0.032	0.113^a^	7(1.6)	9(0.6)	0.835	0.010
**Other comorbidities, *n* (%)**								
aCCI, mean (SD)	5.71(2.1)	5.60(1.9)	0.318	0.053	5.67(2.0)	5.55(1.8)	0.926	0.006
Hypertension	344(75.9)	1091(75.3)	0.847	0.014	332(75.3)	1065(75.0)	0.897	0.008
Hyperlipidemia	181(40.0)	620(42.8)	0.307	0.058	176(39.9)	605(42.6)	0.469	0.047
TD	171(37.7)	360(24.9)	<0.001	0.281^a^	162(36.7)	345(24.3)	0.655	0.027
Vascular disease	16(3.5)	31(2.1)	0.136	0.084	15(3.4)	29(2.0)	0.889	0.007
DM with organ damage	111(24.5)	294(20.3)	0.066	0.101^a^	103(23.4)	283(19.9)	0.527	0.038
CKD	5(1.1)	8(0.6)	0.360	0.061	4(0.9)	7(0.5)	0.908	0.005
CHF	0(0.0)	1(0.1)	1	0.037	0(0.0)	0(0.0)	NA	<0.001
IHD	222(49.0)	637(44.0)	0.069	0.101^a^	215(48.8)	619(43.6)	0.680	0.026
COPD	9(2.0)	36(2.5)	0.665	0.034	9(2.0)	32(2.3)	0.895	0.009
Any tumor	28(6.2)	97(6.7)	0.780	0.021	28(6.3)	89(6.3)	0.737	0.023
CVA	228(50.3)	641(44.3)	0.027	0.122^a^	220(49.9)	619(43.6)	0.668	0.028
Metastatic solid tumor	0(0.0)	2(0.1)	1	0.053	0(0.0)	0(0.0)	NA	<0.001
Leukemia	0(0.0)	2(0.1)	1	0.053	0(0.0)	0(0.0)	NA	<0.001
Hemiplegia	9(2.0)	17(1.2)	0.285	0.065	432(98.0)	9(2.0)	15(1.1)	0.047
Lymphoma	2(0.4)	6(0.4)	1	0.004	2(0.5)	5(0.4)	0.563	0.025
Dementia	0(0.0)	11(0.8)	0.132	0.124^a^	0(0.0)	0(0.0)	NA	<0.001
MLD	20(4.4)	40(2.8)	0.109	0.089	18(4.1)	37(2.6)	0.964	0.002
CTD	2(0.4)	8(0.6)	1	0.016	2(0.5)	8(0.6)	0.892	0.008
Ulcer disease	166(36.6)	381(26.3)	<0.001	0.224^a^	157(35.6)	371(26.1)	0.891	0.008
SLD	0(0.0)	1(0.1)	1	0.037	0(0.0)	0(0.0)	NA	<0.001
AIDS	1(0.2)	0(0.0)	0.539	0.067	0(0.0)	0(0.0)	NA	<0.001

IPTW, inverse probability of treatment weighting; ASMD, absolute standardized mean difference; SGLT2i, sodium-glucose cotransporter-2 inhibitors; SU, sulfonylureas; *n*, number; TZDs, thiazolidinediones; AGI, alpha-glycosidase inhibitors; NSUR, non-sulfonylureas; aDCSI, the adapted diabetes complications and severity index; aCCI, the age-adjusted Charlson comorbidity index; OSAHS, obstructive sleep apnea-hypopnea syndrome; SD, standard deviation; TD, thyroid disease; DM, diabetes mellitus; CKD, chronic kidney disease; CHF, congestive heart failure; IHD, ischemic heart disease; COPD, chronic pulmonary disease; CVA, cerebral vascular disease; MLD, mild liver disease; CTD, connective tissue disease; SLD, moderate or severe liver disease; AIDS, acquired immune deficiency syndrome.

^a^ASMD > 0.1 which indicates a non-negligible difference between the two treatment groups.

After adjusting for IPTW-based propensity scores and excluding the top and bottom 1% of extreme values, there was no significant difference in baseline characteristics between the groups (*n* = 441 for SGLT2i, *n* = 1,420 for SU). ASMD were below 0.1 after weighting, confirming adequate covariate balance and ensuring comparability between the two treatment groups (Table 1; Figure S1).

### SGLT2i reduced the risk of biliary diseases in T2DM patients

**Figure 2.**
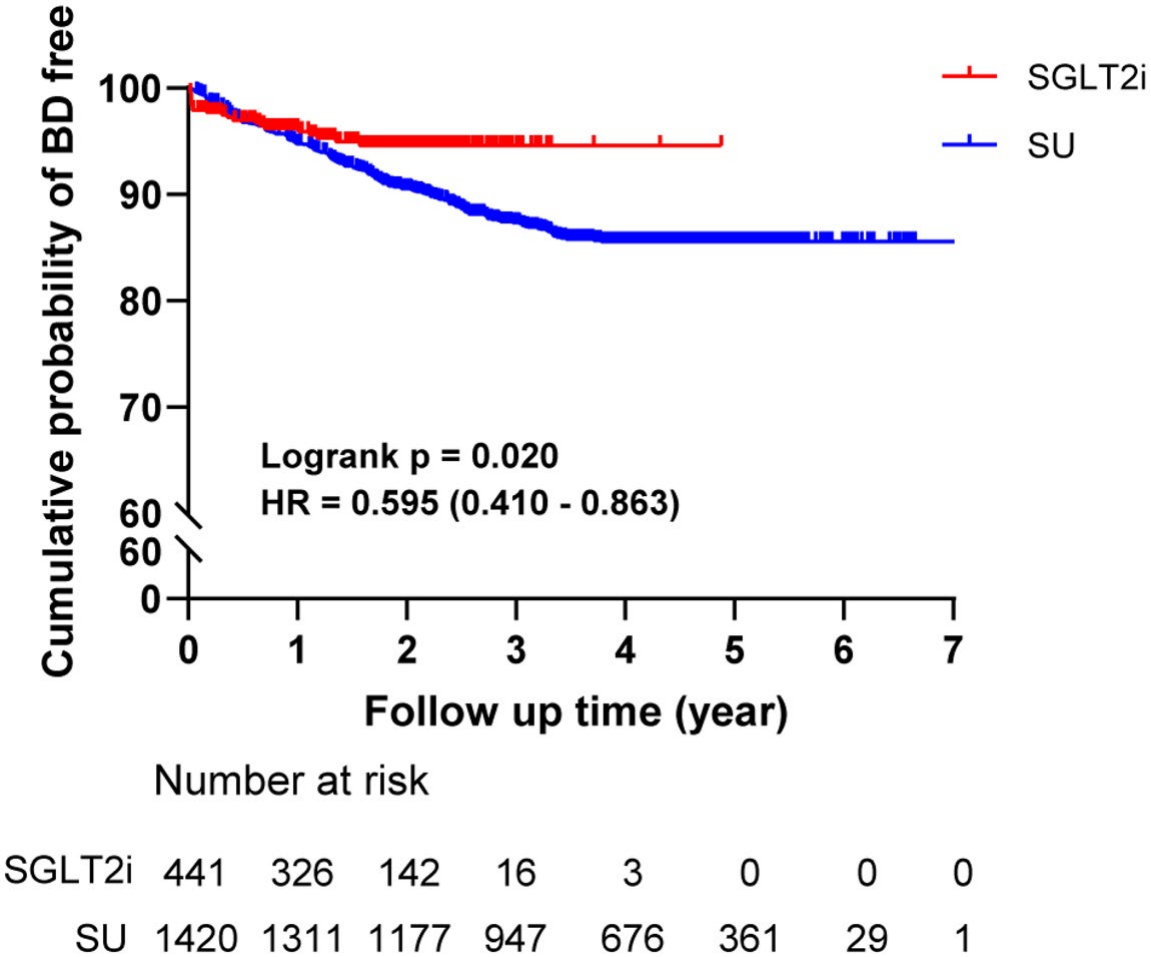
Kaplan–Meier survival curve for BD in two groups.

In the initial unadjusted analysis, patients using SGLT2i exhibited a reduced risk of developing BD (HR, 0.514; 95% CI, 0.311–0.808) in comparison to those starting treatment with sulfonylureas. After excluding the extreme IPTW-adjusted values, the risk remained lower in the SGLT2i group (21 events, 22.7/1000 person-years) relative to the sulfonylurea initiators (187 events, 43.09/1000 person-years), with an HR of 0.595 and 95% CI of 0.410–0.863 (Table 2; Figure 2). Specifically, in the SGLT2i group, there were 10 cases of cholelithiasis, 9 of cholecystitis, 3 of gallbladder polyps, 1 bile reflux, and 1 cholangiocarcinoma, with three patients having both cholelithiasis and cholecystitis. The sulfonylurea group had 101 cases of cholelithiasis, 80 of cholecystitis, 32 polyps, 5 bile reflux, and 3 cholangiocarcinoma, with 34 having both cholelithiasis and cholecystitis (Figure 1).

**Table 2.** Risk of biliary disease for SGLT2i compared to the SU group.

	Patients	Biliary disease	Incidence density (per 1000 person-year)	HR (95% CI)
Crude analysis				
SGLT2i	453	21	21.69	0.514 (0.311–0.808)
SU	1448	189	42.67	Reference
Main analysis				
SGLT2i	441	21	22.70	0.595 (0.410–0.863)
SU	1420	187	43.09	Reference
Sensitivity analysis				
1:1 PSM				
SGLT2i	430	20	22.08	0.552 (0.311–0.945)
SU	430	50	39.97	Reference
1:2 PSM				
SGLT2i	430	20	22.08	0.539 (0.314–0.885)
SU	707	86	40.95	Reference
1:3 PSM				
SGLT2i	430	20	22.08	0.557 (0.327–0.903)
SU	904	108	39.63	Reference
SMRW after 1% removing				
SGLT2i	441	21	22.70	0.595 (0.410–0.863)
SU	1420	187	43.09	Reference
Logistic regression				
SGLT2i	441	.	.	0.379 (0.234–0.614)
SU	1420	.	.	Reference

Crude analysis was performed on 1901 patients while the main analysis was performed on 1861 patients after 1% removal with IPTW.

SGLT2i, sodium-glucose cotransporter-2 inhibitors; SU, sulfonylureas; HR, hazard ratios; CI, confidence intervals; PSM, propensity score matching; SMRW, standardized mortality ratio weighting; IPTW, inverse probability of treatment weighting.

Figure 2 illustrates the cumulative probability of remaining free from biliary diseases for both groups.

### Sensitivity analyses supported the primary analysis

To evaluate the robustness of our primary findings, a series of sensitivity analyses were performed, and all results consistently supported the significant protective association between SGLT2i use and reduced risk of biliary diseases compared with SU.

First, multiple PSM strategies were applied, including 1:1, 1:2, and 1:3 matching ratios. Across all matching schemes, the HR remained consistently below 0.56, with statistically significant 95% CI (1:1 PSM, HR = 0.552, 95% CI: 0.311–0.945; 1:2 PSM, HR = 0.539, 95% CI: 0.314–0.885; 1:3 PSM, HR = 0.557, 95% CI: 0.327–0.903), indicating a robust association regardless of matching strategy. Second, SMRW analysis was conducted after removing the top and bottom 1% of outliers based on propensity scores. This approach also yielded a consistent result (HR = 0.595, 95% CI: 0.410–0.863), reaffirming the reliability of the main analysis. Third, a logistic regression model was employed to further validate the findings, producing an even stronger association (HR = 0.379, 95% CI: 0.234–0.614), further strengthening the evidence of a protective effect.

Together with the crude and main analyses (HR of 0.514 and 0.527, respectively), these sensitivity tests underscore the robustness and stability of the observed association between SGLT2i use and a lower incidence of BD. Detailed results from these analyses are presented in Table 2.

### Additional comparator analysis for sulfonylureas

To assess whether sulfonylureas itself promoted BD, we compared SU users with an active-treated comparator cohort exposed to neither SU nor SGLT2i. Baseline characteristics were broadly comparable between groups (Table S4). The crude incidence density was 42.67 and 42.71 per 1,000 person-years in the SU and comparator groups, respectively (IRR 0.999, 95% CI 0.842–1.186; *p* = 0.993). Consistent results were observed in regression analyses, with no evidence of increased BD risk associated with SU (unadjusted Cox: HR 0.908, 95% CI 0.765–1.078; *p* = 0.270; adjusted Cox: HR 0.870, 95% CI 0.732–1.033; *p* = 0.112; adjusted logistic regression: OR 0.893, 95% CI 0.740–1.075; *p* = 0.235) (Table S5).

### Risk of biliary diseases over time

To further explore the temporal dynamics of SGLT2i’s protective effect, a time-stratified analysis was conducted at 6, 12, 18, and 24 months of follow-up (Table 3). While no statistically significant difference in the incidence of biliary disease was observed during the first 6 months (HR = 0.912, 95% CI: 0.475–1.632, *p* = 0.751) or at 12 months (HR = 1.028, 95% CI: 0.559–1.784, *p* = 0.919), the hazard ratio progressively decreased over time. By 18 months, a protective trend was noted (HR = 0.700, 95% CI: 0.414–1.125, *p* = 0.129), and this became statistically significant at 24 months. At that time point, the incidence rate of biliary diseases in the SGLT2i group was 22.70 per 1,000 person-years compared to 36.88 in the SU group, with a hazard ratio of 0.616 (95% CI: 0.371–0.974, *p* = 0.032), indicating a 38.4% reduced risk of biliary disease in the SGLT2i group compared to the SU group. Importantly, the overall trend was statistically significant (*p* for trend = 0.028), suggesting a cumulative benefit of SGLT2i with longer duration of use.

**Table 3.** Risk of biliary disease for SGLT2i compared with SU group at different time stages.

	Patients	Disease	Incidence rate (per 1000 person-year)	HR (95%CI)	*p*
**Outcomes at 6 months**					
**SGLT2i**	441	14	15.14	0.912(0.475–1.632)	0.751
**SU**	1420	72	17.05	Reference	
**Outcomes at 12 months**					
**SGLT2i**	441	16	17.30	1.028(0.559–1.784)	0.919
**SU**	1420	73	16.82	Reference	
**Outcomes at 18 months**					
**SGLT2i**	441	20	21.62	0.700(0.414–1.125)	0.129
**SU**	1420	134	30.88	Reference	
**Outcomes at 24 months**					
**SGLT2i**	441	21	22.70	0.616(0.371–0.974)	0.032*
**SU**	1420	160	36.88	Reference	
***p* for trend**					0.028*

SGLT2i, sodium-glucose cotransporter-2 inhibitors; SU, sulfonylureas; HR, hazard ratios; CI, confidence intervals.

*The asterisk indicates a statistically significant difference.

Together, these complementary analyses strengthen the credibility of the main results and underscore the potential of SGLT2i in long-term biliary disease risk reduction.

### Subgroup analyses revealed consistent and enhanced protective effects in specific populations

Subgroup analyses revealed notable heterogeneity in the effect of SGLT2i on biliary disease risk across different patient profiles (Table 4). A significantly reduced risk was observed among patients aged over 60 years (HR = 0.490, 95% CI: 0.229–0.934), as well as in those without diabetic complications (aDCSI = 0, HR = 0.432, 95% CI: 0.169–0.929). Interestingly, patients with a higher comorbidity burden (aCCI ≥ 5) also benefited markedly (HR = 0.419, 95% CI: 0.204–0.774), suggesting that SGLT2i may provide added protection in clinically vulnerable populations. In addition, when stratified by prior biguanides (metformin) exposure, SGLT2i use remained associated with a significantly lower risk of biliary diseases in both metformin users (HR = 0.465, 95% CI: 0.259–0.781) and non-users (HR = 0.249, 95% CI: 0.098–0.530), indicating that the observed protective effect was independent of background biguanides therapy.

**Table 4.** Risk of biliary disease for SGLT2i compared with SU group in subgroups analyses.

	Subgroups									
	*N*	Events	SGLT2i#	SU#	HR95%CI (RF:SU)	*N*	Events	SGLT2i#	SU#	HR95%CI (RF:SU)
	age ≤ 60years					>60years				
All	788	84	23.86	41.72	0.572(0.274–1.085)	1073	124	21.55	44.01	0.490(0.229–0.934)
	aDCSI = 0(without diabetic complications)					aDCSI > 0(with at least one diabetic complication)				
All	801	94	19.07	44.13	0.432(0.169–0.929)	1060	114	25.09	42.22	0.594(0.314–1.044)
	aCCI <5(low group)					aCCI ≥5 (high group)				
All	562	63	31.25	42.26	0.739(0.335–1.469)	1299	145	18.18	43.42	0.419(0.204–0.774)
	Treated with canagliflozin					Treated with dapagliflozin or empagliflozin				
All	55	7	7.57	43.09	0.176(0.070–0.369)	386	14	15.14	43.09	0.351(0.188–0.604)
	Treated with biguanides					Treated without biguanides				
All	1338	157	21.77	46.85	0.465(0.259–0.781)	523	122	9.52	38.21	0.249(0.098–0.530)

# incidence rate per 1000 person-years.

SGLT2i, sodium-glucose cotransporter-2 inhibitors; SU, sulfonylureas; HR, hazard ratios; CI, confidence intervals; aDCSI, the adapted diabetes complications and severity index; aCCI, the age-adjusted charlson comorbidity index; RF, reference group.

In terms of specific agents, treatment with canagliflozin showed the strongest association with risk reduction (HR = 0.176, 95% CI: 0.070–0.369), while dapagliflozin or empagliflozin users also demonstrated significant benefits (HR = 0.351, 95% CI: 0.188–0.604). These consistent findings across drug types further support the robustness of the protective effect of SGLT2i against biliary disease.

### No significant differences in weight, body mass index (BMI), FPG, and HbA1c between groups

**Figure 3.**
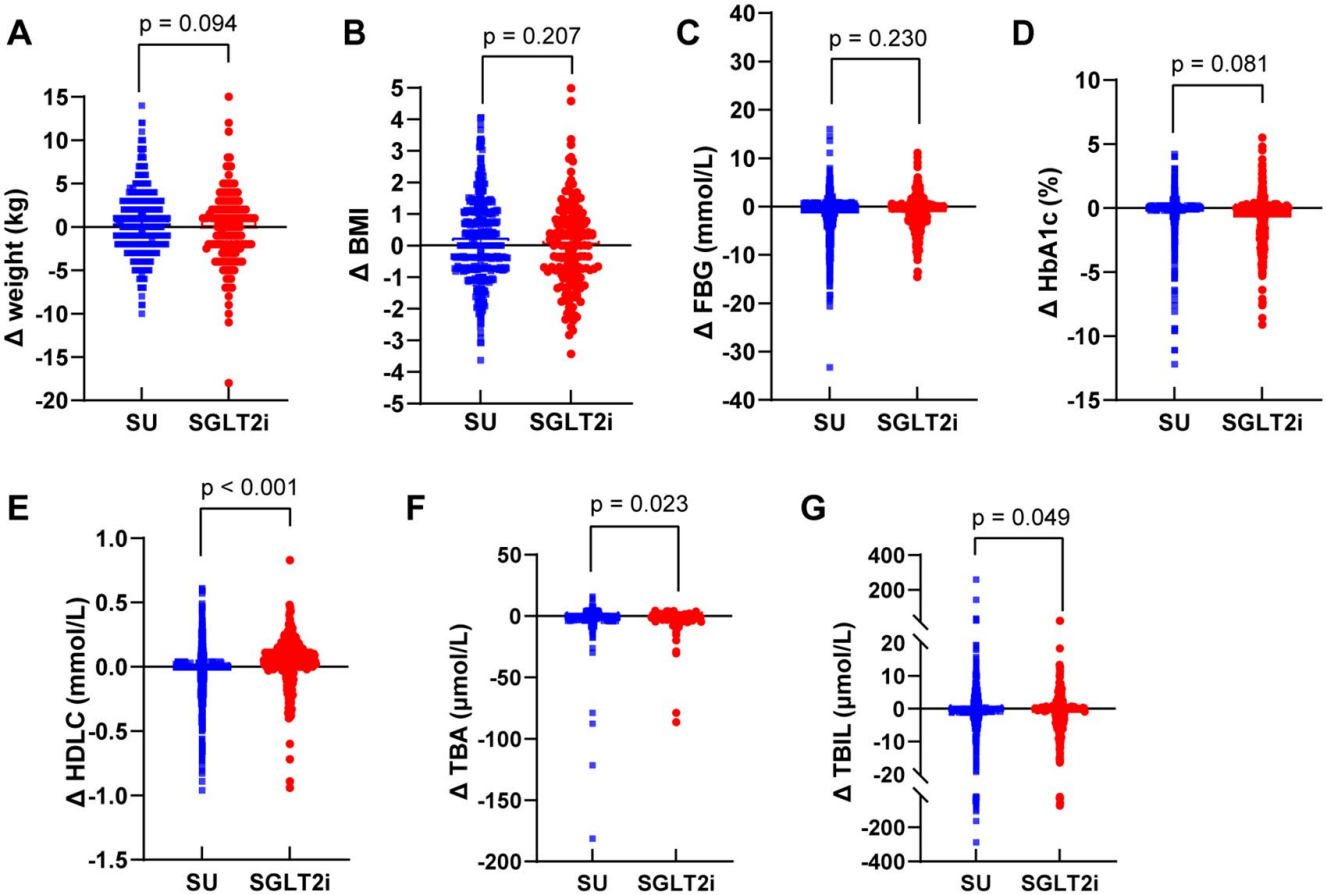
Effect of SGLT2i on the clinical index in T2DM. (A) Change of weight (kg) between the two groups. (B) Change of BMI (kg/m^2^) between the two groups. (C) Change of serum FBG levels between the two groups. (D) Change of serum HbA1c levels between the two groups. (e) Change of serum HDLC levels between the two groups. (F) Change of serum TBA levels between the two groups. (G) Change of serum TBIL levels between the two groups. All changes were estimated from the index date to the termination date of the follow-up; *p < 0.05, **p < 0.01; ***p < 0.001.

At baseline, no significant differences in body weight or BMI were observed between the SGLT2i and SU groups. The median [interquartile range (IQR)] weight was 69 [60, 80] kg in the SGLT2i group and 69 [64, 76] kg in the SU group, with mean BMI values of 25.30 and 25.36 kg/m^2^, respectively (Table 5). During follow-up, changes in weight and BMI were minimal and remained comparable. At the end of the study, both groups maintained similar values (SGLT2i group: 69 [61.75, 78] kg, BMI = 25.28 kg/m^2^; SU group: 70 [64, 76] kg, BMI = 25.50 kg/m^2^; Table 5 and Figure 3A,B), indicating no significant treatment effect on weight-related outcomes.

**Table 5.** Differences in laboratory examinations between the two treatment groups in the index date and the termination date of follow-up.

	SGLT2i	*p*1	SU	*p*2	*t/Z*	*p*
Weight, M(IQR), kg		0.529		0.062		
Index date	69(60,80)		69(64,76)		−0.319	0.750
Termination date	69(61.75,78)		70(64,76)		−0.787	0.431
ΔWeight, M(IQR), kg	0(−2.50,2.00)	–	0(−2.00,3.00)	–	1.676	0.094
BMI, mean (SD), kg/m^2^		0.605		0.056		
Index date	25.30(3.53)		25.36(2.86)		−0.505	0.614
Termination date	25.28(2.69)		25.50(2.37)		−1.273	0.203
ΔBMI, mean (SD), kg/m^2^	0(−0.94,0.81)	–	0(−0.73,1.01)	–	1.263	0.207
FBG, M(IQR), mmol/L		0.000		0.000		
Index date	7.81(6.31,10.34)		7.64(5.86,10.50)		−0.577	0.564
Termination date	6.80(5.83,8.50)		6.94(5.52,8.71)		−0.107	0.915
ΔFBG, M(IQR), mmol/L	−0.07(−2.70,0)	–	0(−2.01,0)	–	1.202	0.230
HbA1c, mean (SD), %		0.000		0.000		
Index date	8.18(1.78)		7.89(2.10)		2.279	0.023*
Termination date	7.44(1.57)		7.61(2.00)		−1.506	0.132
ΔHbA1c, mean (SD), %	−0.28(1.51)	–	−0.49(1.51)	–	1.749	0.081
TC, M(IQR), mmol/L		0.021		0.000		
Index date	4.41(3.70,5.16)		4.41(3.76,5.19)		−0.076	0.939
Termination date	4.32(3.53,5.27)		4.29(3.59,5.09)		−0.733	0.464
TG, M(IQR), mmol/L		0.000		0.000		
Index date	1.58(1.09,2.46)		1.52(1.06,2.21)		−1.659	0.097
Termination date	1.43(1.05,1.93)		1.44(1.02,2.03)		−0.17	0.865
LDLC, mean (SD), mmol/L		0.093		0.000		
Index date	2.65(0.89)		2.67(0.93)		−0.425	0.671
Termination date	2.58(0.94)		2.55(0.94)		0.483	0.629
HDLC, mean (SD), mmol/L		0.000		0.000		
Index date	1.08(0.20)		1.11(0.29)		−1.852	0.064
Termination date	1.13(0.22)		1.08(0.30)		3.121	0.002***
ΔHDLC, mean (SD), mmol/L	0.03(0.17)	–	−0.02(0.19)	–	4.433	0.000***
LPa, M(IQR), mg/L		0.002		0.512		
Index date	42(15.82,150)		91(29.75,203)		−4.308	0.000***
Termination date	37(12,94)		89.2(28.52,194)		−6.715	0.000***
TBA, M(IQR), μmol/L		0.000		0.011		
Index date	3.40(2.40,6.02)		3.80(2.40,6.20)		−0.693	0.488
Termination date	2.60(2.10,4.13)		3.70(2.40,5.85)		−5.064	0.000***
ΔTBA, M(IQR), μmol/L	−0.80(−2.12,−0.10)	–	0(0,0.20)	–	2.272	0.023*
TBIL, M(IQR), μmol/L		0.000		0.052		
Index date	12.59(9.30,16.40)		12.70(9.68,16.50)		−0.836	0.403
Termination date	11.64(8.80,15.60)		12.49(9.30,16.60)		−2.459	0.014*
ΔTBIL, M(IQR), μmol/L	0(−3.72,0)	–	0(−0.90,0.30)	–	1.966	0.049*
DBIL, M(IQR), μmol/L		0.001		0.007		
Index date	4.22(3.40,5.60)		4.33(3.20,5.70)		−0.365	0.715
Termination date	4.10(3.10,5.40)		4.20(3.12,5.70)		−1.503	0.133
IBIL, M(IQR), μmol/L		0.002		0.436		
Index date	7.40(5.17,10.30)		7.30(5.10,9.99)		−0.530	0.596
Termination date	6.85(4.98,9.60)		7.20(5.10,9.80)		−0.953	0.341

FPG, fasting plasma glucose; HbA1c, glycosylated hemoglobin; SGLT2i, sodium-glucose cotransporter-2 inhibitors; SU, sulfonylureas; TC, total cholesterol; TG, triglyceride; LDLC, low density lipoprotein cholesterol; HDLC, high density lipoprotein cholesterol; LPa, lipoprotein a; TBA, total bile acid; TBIL, total bilirubin; DBIL, direct bilirubin; IBIL, indirect bilirubin; SD, standard deviation; *M* (IQR), median (interquartile range); *N*, number. Δ represents the change between the termination date and the index date.

The analysis was performed on 1861 patients after 1% removal with IPTW. *p* value is the comparison of clinical characteristics in SGLT2i group and SU group both in index date and termination date. The *p*1 value compares the change in clinical features from the index date to the termination date in the SGLT2i group, while *p*2 is in the SU group.

*The asterisk indicates a statistically significant difference. *p < 0.05, **p < 0.01, ***p < 0.001.

Similarly, FPG and HbA1c levels improved modestly in both groups, but without significant intergroup differences. Initial mean (SD) HbA1c levels were 8.18 (1.78)% in the SGLT2i group and 7.89 (2.10)% in the SU group. By follow-up, these levels decreased to 7.44 (1.57)% and 7.61 (2.00)%, respectively (Table 5; Figure 3C,D). These findings suggest that the observed biliary protection associated with SGLT2i is unlikely to be mediated by weight loss or glycemic control.

### Significant improvements in lipid profile and biliary biomarkers in the SGLT2i group

Both treatment groups experienced significant reductions in TC and TG during follow-up. In the SGLT2i group, TC decreased from 4.41 [3.70, 5.16] to 4.32 [3.53, 5.27] mmol/L (*p* = 0.021), and TG from 1.58 [1.09, 2.46] to 1.43 [1.05, 1.93] mmol/L (*p* < 0.001). Similar reductions were seen in the SU group (TC: from 4.41 [3.76, 5.19] to 4.29 [3.59, 5.09], *p* < 0.001; TG: from 1.52 [1.06, 2.21] to 1.44 [1.02, 2.03], *p* < 0.001), with no significant difference in the magnitude of lipid-lowering effects. However, the SGLT2i group showed a significant increase in HDLC (*p* = 0.002), a change not observed in the SU group. Moreover, serum TBA and TBIL levels decreased significantly in the SGLT2i group compared to the SU group (TBA: *p* < 0.001; TBIL: *p* = 0.014; Table 5; Figure 3E–G). Specifically, median TBA levels decreased by 0.80 μmol/L (from 3.40 [2.40–6.02] to 2.60 [2.10–4.13] μmol/L) in the SGLT2i group, whereas they remained essentially unchanged in the SU group (3.80 [2.40–6.20] to 3.70 [2.40–5.85] μmol/L). Similarly, TBIL decreased by a median of 0.95 μmol/L (from 12.59 [9.30–16.40] to 11.64 [8.80–15.60] μmol/L) in the SGLT2i group but showed minimal change in the SU group (12.70 [9.68–16.50] to 12.49 [9.30–16.60] μmol/L). These specific improvements in biliary biomarkers may partially explain the observed reduction in biliary disease risk among SGLT2i users, independent of glucose and weight control.

## Discussion

In this large real-world cohort, we found that the use of SGLT2i was associated with a significantly lower risk of BD in patients with T2DM compared with sulfonylureas. This protective association became more evident with longer treatment duration and was particularly pronounced in patients aged over 60 years, those without diabetic complications, and those with a higher comorbidity burden (aCCI ≥ 5). Notably, the observed protective effect was independent of glycemic control and weight reduction, indicating that SGLT2i may act through mechanisms beyond conventional metabolic regulation.

The prevalence and incidence of gallstones were 5 − 25%, and 0.47 per 100 person-years, respectively. Diabetes significantly increases the risk of gallbladder disease, with a prevalence of up to 33.3% among diabetics. A meta-analysis of ten cohort studies indicated the relative risk of gallbladder disease among diabetic patients was 1.56 (1.26–1.93) compared to non-diabetic. BD can cause severe complications like bile duct obstruction and pancreatitis, exacerbating T2DM outcomes. Moreover, surgical interventions like cholecystectomy pose additional risks for T2DM patients, including delayed wound healing and higher infection rates. Preventing BD is therefore crucial. While the cardiovascular and renal benefits of SGLT2i are well established [7–12], the impact of SGLT2i on biliary outcomes has remained poorly characterized. Our findings fill this knowledge gap by demonstrating that SGLT2i use is associated with a substantially lower incidence of BD compared to sulfonylureas.

Cholesterol cholelithiasis, influenced by genetic and environmental factors, has a controversial link to blood glucose [29]. Despite achieving glycemic targets, many patients still develop gallstones [5,29], indicating hyperglycemia may not be an independent risk factor. The observed BD prevention benefits of SGLT2i seem unrelated to glucose or weight reduction, as weight, BMI, FPG, and HbA1c changes were similar between groups. Cholesterol stone formation, which accounts for over 90% of biliary stones, involves cholesterol supersaturation and gallbladder hypomotility [30]. Lowering hepatic cholesterol reduces its secretion into bile. Several biological mechanisms may underlie the observed biliary benefits. In our study, the SGLT2i group showed significant reductions in serum TBA and TBIL, along with an increase in HDLC, indicating improvements in bile flow, cholesterol handling, and liver function. These changes may collectively contribute to reduced biliary stasis and BD formation.

Recent preclinical studies have provided additional insight into the hepatic metabolic effects of SGLT2 inhibitors. SGLT2i have been shown to activate the AMPK-SIRT1 signaling pathway, enhancing autophagy, reducing hepatic lipid accumulation, alleviating hepatic fibrosis, normalizing bile acid levels, reducing ER stress and inflammation and improving overall metabolic homeostasis [14,31]. Moreover, recent experimental studies further demonstrated that empagliflozin alleviates hepatic steatosis and oxidative stress *via* the NRF1 pathway in a high-fat diet-induced metabolic dysfunction-associated steatotic liver disease model, reducing ER and oxidative stress and improving mitochondrial function [32]. Another study using a prediabetic rat model found that empagliflozin improved hepatic lipid metabolism independently of obesity and hyperglycemia, mainly through decreased lipogenesis, altered cytochrome P450 enzyme expression, and reduced fetuin-A levels [33]. These findings consistently support that SGLT2 inhibitors improve hepatic lipid homeostasis and oxidative balance through multiple metabolic and transcriptional pathways, providing mechanistic plausibility for their observed biliary and hepatic protective effects. However, direct experimental evidence on how SGLT2i modulate biliary physiology remains scarce. In our ongoing animal experiments (unpublished), SGLT2i treatment improved gallbladder emptying fraction, promoted bile acid synthesis, and favorably modified the bile acid profile, suggesting that modulation of bile acid metabolism may contribute to the clinical association observed in this study. These preliminary findings provide a biological basis for further mechanistic investigation into the hepatobiliary effects of SGLT2 inhibitors. In addition, recent evidence indicates that SGLT1, rather than SGLT2, is expressed in cholangiocytes and may facilitate glucose reabsorption from bile [34]. Although most SGLT2 inhibitors primarily act at the renal level, some agents (such as canagliflozin), exhibit partial SGLT1 inhibitory activity, which could influence biliary glucose handling and bile volume. While this off-target mechanism remains speculative, it may provide an additional biological explanation for the observed differences among specific SGLT2 inhibitors.

Moreover, emerging evidence highlights that all SGLT2 inhibitors exert inhibitory activity to varying degrees on both SGLT1 and SGLT2 targets, and SGLT1-mediated mechanisms may contribute to their extra-glycemic benefits. Recent preclinical work by Liang et al. [31], demonstrated that sotagliflozin (a dual SGLT1/2 inhibitor) markedly improved liver function and bile acid metabolism in a cystic fibrosis (CF)-associated liver disease (CFLD) rabbit model. The treatment normalized serum bile acid parameters, alleviated hepatic fibrosis and non-alcoholic steatohepatitis (NASH)-like phenotypes, and reduced endoplasmic reticulum (ER) stress and inflammatory signaling in hepatocytes. Importantly, this study also observed downregulation of hepatic SGLT1 expression after sotagliflozin therapy, suggesting that pharmacological blockade of SGLT1 may protect against biliary and metabolic stress by interrupting a pathological cycle involving CF-induced ER stress, SGLT1 upregulation, and subsequent hepatic injury. These findings further support that partial SGLT1 inhibition may mediate hepatobiliary protection and could partly explain the observed differences among SGLT2i with distinct selectivity profiles.

Nevertheless, the pronounced risk reduction observed with canagliflozin should be interpreted with caution. This estimate was derived from a subgroup analysis with a limited number of events, which may have led to statistical variability and an unstable hazard ratio. Therefore, this finding should not be overinterpreted as a definitive drug-specific effect but rather considered a potential chance observation. Larger, prospective studies are warranted to confirm whether this difference reflects a true agent-specific property or a class-wide effect among SGLT2i.

By the way, we selected sulfonylureas as the comparator group based on their common use in prior studies [35,36], overlapping treatment guidelines, and comparable baseline characteristics, which together provided a clinically relevant and methodologically stable reference. According to the 2024 ADA consensus report, sulfonylureas are no longer recommended as first-line therapy but remain a widely used second-line option in real-world clinical practice, particularly in cost-sensitive settings and in patients who cannot tolerate newer agents [17]. Importantly, to address the concern that the observed association might be driven by a potential SU-related increase in BD risk, we additionally compared SU users with an active-treated cohort exposed to neither SU nor SGLT2i. This analysis did not suggest an increased BD risk associated with SU across crude incidence rates, Cox proportional hazards models, and logistic regression. Therefore, the primary findings are unlikely to be explained by SU-promoted biliary disease. In addition, we also performed a subgroup analysis stratified by prior metformin exposure, rather than using metformin as a separate comparator group. The results showed that SGLT2i use was associated with a lower risk of biliary diseases compared with sulfonylureas in both patients with and without prior metformin use, indicating that the observed association was not confounded by background metformin therapy. This additional analysis strengthens the robustness of our findings while underscoring the need for future mechanistic and prospective studies to clarify the causal relationship. Notably, the lower incidence of biliary diseases among patients treated with SGLT2i may help inform therapeutic strategies for individuals at elevated risk, particularly elderly patients.

Although some pharmacovigilance data have suggested a possible signal between SGLT2i use and pancreatitis [37,38], our findings do not support this concern. In the present cohort, no cases of acute pancreatitis occurred during the follow-up period, and patients with a prior history of pancreatitis were excluded at baseline. Given that pancreatitis and biliary diseases share overlapping anatomical and metabolic pathways within the pancreatic–biliary system, it is reassuring that the observed association between SGLT2i use and lower biliary disease risk was not confounded by pancreatic events. Nevertheless, pancreatic safety remains an important consideration, and we will continue to monitor and evaluate potential pancreatic effects of SGLT2i in future studies.

To the best of our knowledge, this study has several strengths. It is the first to systematically investigate the association between SGLT2i use and BD risk in patients with T2DM using real-world data from a multicenter database, applied rigorous confounding adjustment *via* multiple propensity score methods, and validated findings through time-stratified and subgroup analyses. As one of its advantages, our cohort study with active comparator new-users was designed to minimize selection bias and exclude numerous relevant confounding factors, resulting in a more representative selected population. Moreover, the observed benefits extend the therapeutic relevance of SGLT2i beyond their current indications, highlighting their potential for drug repurposing. Given their established safety profile and pleiotropic effects, SGLT2i may offer a valuable, efficient treatment option for patients with multimorbidity, with broader implications for reducing disease burden and healthcare costs.

This study also has several limitations. First, as a retrospective cohort study, it lacked certain parameters, such as patient serum insulin levels, preventing a definitive conclusion on whether SGLT2i reduces BD incidence by improving insulin resistance.

Second, as an observational study, potential residual confounding, selection bias, and information bias cannot be entirely excluded despite the use of robust statistical adjustments such as IPTW and multiple sensitivity analyses. Information on dietary habits, gallbladder motility, imaging confirmation and concomitant medication use, which may influence bile composition and gallbladder function, was not available in the electronic health records. Although information on specific BD subtypes (such as cholelithiasis, cholangitis, and others) was included in our dataset, the number of cases within each subtype was limited, reducing the statistical power for reliable individual analyses. Therefore, a composite outcome encompassing multiple BD was adopted to ensure sufficient event numbers and analytical robustness. The reliance on ICD-9/10 codes without universal image-based validation may have introduced potential misclassification or missing data, which is an inherent limitation of electronic health record-based studies. Moreover, the use of a composite outcome encompassing various biliary diseases may also obscure potential disease-specific effects, as the underlying mechanisms differ across these conditions. Furthermore, the long-term effects of SGLT2i on BD beyond the current follow-up duration require further investigation. Third, premenopausal women were excluded from this analysis to minimize hormonal confounding; however, this limits the generalizability of the findings to the overall female population. In addition, the marked risk reduction observed with canagliflozin should be interpreted with caution, as this estimate was derived from a subgroup analysis with a limited number of events, which may have led to statistical variability. Larger, prospective studies are needed to verify whether this reflects a true drug-specific effect or a class-wide feature of SGLT2i. Finally, this study only provides a preliminary exploration of the possible mechanisms underlying the association between SGLT2i and a reduced risk of BD. Further detailed validation is necessary. Therefore, additional prospective long-term and mechanistic studies are warranted to elucidate the causal pathways and confirm these findings. Future studies should also analyze specific BD subtypes separately, incorporate detailed data on lifestyle and medication factors, and include a broader range of female participants to confirm the observed associations, while continuing to investigate potential drug-related effects on biliary and pancreatic safety to improve the generalizability of these findings.

In conclusion, our findings suggest that SGLT2i use is associated with a reduced risk of BD in patients with T2DM, particularly with prolonged use and among high-risk subgroups. These benefits appear to extend beyond glycemic control and may be mediated by favorable effects on bile acid metabolism and liver function. Future randomized controlled trials and mechanistic studies are warranted to validate these findings and explore the potential of SGLT2i in BD prevention.

## Conclusion

SGLT2i use was associated with a lower risk of BD in T2DM patients, independent of glycemic or weight-related effects. The association appeared stronger with prolonged treatment duration and among high-risk subgroups. These findings indicate a potential biliary benefit of SGLT2i but should be interpreted with caution. Further large-scale prospective and mechanistic studies are warranted to confirm these results and clarify the underlying biological pathways.

## Supplementary Material

clean updated supplementary materials.docx

## Data Availability

The data that support the findings of this study are available from the corresponding author upon reasonable request.

## Preprint notice

A preliminary version of this study, including additional animal and cellular experiments alongside the retrospective cohort data, was previously posted as a preprint (DOI: https://doi.org/10.1101/2024.10.15.24315461]) published on medRxiv [39]. The present manuscript focuses solely on the clinical cohort study.
