# Supplementary material for: Association of SGLT2 inhibitors use with a lower risk of biliary diseases in patients with type 2 diabetes mellitus: a retrospective cohort study
Source: Ann Med. 2026 Feb 2;58(1):2619263. doi: 10.1080/07853890.2026.2619263 (PMC12865853; doi:10.1080/07853890.2026.2619263)
Supplement: clean updated supplementary materials.docx [file IANN_A_2619263_SM0408.docx]

**Supplementary Tables**

**Table S1.** Diagnosis codes for all diseases in the study.

**Table S2.** Age-adjusted Charlson Comorbidity Index.

**Table S3.** Individual drug for study medication.

**Table S4.** Baseline characteristics of SU group and Neither SU nor SGLT2i (active-treated comparator) group.

**Table S5.** Association between sulfonylurea use and BD risk in the SU group and the Neither SU nor SGLT2i (active-treated comparator) group.

**Supplementary Figures**

**Figure S1.** The distribution of variables after IPTW.

**Table S1. Diagnosis codes for all diseases in the study**

|  | **ICD-9/10-CM** |
| --- | --- |
| Type 2 diabetes mellitus | E11 |
| Diabetes retinopathy | E113 |
| Diabetes neuropathy | E114 |
| Diabetes nephropathy | E112 |
| Stroke | E13.500x241+I79.2* |
| Metabolic emergency | E110, E111 |
| Cardiovascular disease | E11.502+I79.2* |
| Obstructive sleep apnea-hypopnea syndrome | G47.300x037, G47.301 |
| Hypertension | I10-I16 |
| Hyperlipidemia | E78 |
| Thyroid disease | E01-E07 |
| Vascular disease | I51.600, I67.800, I73.800, I73.900, I78.800, I78.900 |
| Diabetes with organ damage | E112-E117 |
| Chronic kidney disease | N18.800 |
| Congestive heart failure | I50 |
| Ischemic heart disease | E11.500x031+I43.8*, I25.500 |
| Chronic obstructive pulmonary  disease | J44 |
| Any tumor | C00-D48 |
| Cerebral vascular disease | I67.800, |
| Metastatic solid tumor | M80000/6 |
| Leukemia | C91-C95 |
| Hemiplegia | G81 |
| Lymphoma | C81-C88 |
| Dementia | F03 |
| Mild liver disease | K72.905 |
| Connective tissue disease | M35 |
| Ulcer disease | K25, K26, K28 |
| Moderate or severe renal disease | N19.x01, |
| Moderate or severe liver disease | K72.1, K70.300, K74.100, |
| Acquired immune deficiency syndrome | B22, B23, B24 |
| Biliary stones | K80, K80.0, K80.1, K80.2, K80.3, K80.4, K80.5, K80.8 |
| Cholecystitis | K81, K81.0, K81.1, K81.8, K81.9 |
| Other diseases of the gallbladder | K82, K82.0, K82.1, K82.2, K82.3, K82.4, K82.8, K82.9 |
| Other diseases of biliary tract | K83, K83.0, K83.1, K83.2, K83.3, K83.4, K83.5, K83.8, K83.9 |
| Biliary acute pancreatitis | K85.0 |
| Diseases of gallbladder and biliary tract caused by diseases classified elsewhere | K87*, K87.1* |
| Biliary cirrhosis | K74.3, K74.4, K74.5 |
| Cholangiocarcinoma | C22.1, C24.0 |

Abbreviation: ICD-9/10-CM, International Classification of Diseases, Ninth or Tenth Revision, Clinical Modification.

**Table S2. Age-adjusted Charlson Comorbidity Index**

|  | **Conditions** |
| --- | --- |
| Assigned weights for disease |  |
| 1 | Myocardial infarction (MI)  Congestive heart failure (CHF)  Peripheral vascular disease (PVD)  Cerebrovascular disease  Dementia  Chronic pulmonary disease (COPD)  Connective tissue disease (CTD)  Ulcer disease  Mild liver disease (MLD)  Diabetes |
| 2 | Hemiplegia  Moderate or severe renal disease  Diabetes with endo organ damage  Any tumor  Leukemia  Lymphoma |
| 3 | Moderate or severe liver disease (SLD) |
| 6 | Metastatic solid tumor  Acquired immune deficiency syndrome (AIDs) |
| Assigned weights for age |  |
| 1 | For each decade over age 40 years (up to 4 points) |

**Table S3. Individual drugs for study medication**

| **Drug class** | **Generic name of drug** |
| --- | --- |
| Sodium-dependent glucose transporters 2 inhibitors | Canagliflozin, Empagliflozin, Dapagliflozin |
| Sulfonylureas | Glipizide, Gliclazide, Gliquidone, Glimepiride, Glyburide |
| Non-sulfonylureas | Repaglinide, Mitiglinide, Nateglinide |
| Biguanides | Metformin |
| Thiazolidinediones | Troglitazone, Rosiglitazone, Pioglitazone |
| Alpha-glycosidase inhibitors | Acarbose, Miglitol, Voglibose |
| Dipeptidyl peptidase-4 inhibitors | Sitagliptin, Vildagliptin, Saxagliptin, Alogliptin, Linagliptin |
| Glucagon-like peptide-1 receptor agonists | Semaglutide, Dulaglutide, Exenatide, Liraglutide, Loxenatide |
| Insulins | Rapid insulins, Short insulins, Intermediate insulin, Long-acting insulins or insulin analogue |

**Table S4.** Baseline characteristics of SU group and Neither SU nor SGLT2i (active-treated comparator) group.

|  | **SU** | **Neither SU nor SGLT2i** | **p** |
| --- | --- | --- | --- |
| n | 1448 | 3054 | - |
| Sex, Male, n (%) | 920 (63.54) | 1914 (62.67) | 0.597 |
| Age, mean (SD) | 61.95 (9.35) | 62.19 (10.29) | 0.443 |
| Smoke, n (%) | 569 (39.30) | 1280 (41.91) | 0.098 |
| Drink, n (%) | 474 (32.73) | 1026 (33.59) | 0.588 |
| BMI, mean (SD), kg/m^2^ | 25.20 (2.18) | 25 (2.49) | 0.088 |
| FBG, mean (SD), mmol/L | 7.94 (4) | 7.97 (2.88) | 0.820 |
| HbA1c, mean (SD), % | 8.16 (2.18) | 8.17 (1.97) | 0.943 |
| aCCI, mean (SD) | 5.60 (1.89) | 5.52 (1.85) | 0.145 |
| aDCSI≥1, n(%) | 809 (55.87) | 1650 (54.03) | 0.249 |
| Antidiabetic medications, n(%)  Insulin  Biguanides  TZDs  AGI  NSUR | 637 (43.99)  1014 (70.03)  99 (6.84)  690 (47.65)  82 (5.66) | 1272 (41.65)  2107 (68.99)  208 (6.81)  1460 (47.81)  184 (6.02) | 0.138  0.533  0.974  0.924  0.685 |

All patients in this supplementary analysis were selected from the same source population and satisfied the original eligibility criteria, baseline assessment window, and outcome ascertainment rules used in the primary cohort. The SU group included sulfonylurea-treated patients with no SGLT2i exposure before or after the index date. The “Neither SU nor SGLT2i” (active-treated comparator) group included patients receiving other glucose-lowering therapies but with no exposure to sulfonylureas or SGLT2i throughout follow-up. Continuous variables are presented as mean (SD) and categorical variables as n (%). Abbreviations: SU, sulfonylureas; SGLT2i, sodium-glucose cotransporter-2 inhibitors; TZDs, thiazolidinediones; AGI, alpha-glucosidase inhibitors; NSUR, non-sulfonylurea insulin secretagogues; aDCSI, adapted Diabetes Complications Severity Index; aCCI, age-adjusted Charlson Comorbidity Index; SD, standard deviation.

**Table S5.** Association between sulfonylurea use and BD risk in the SU group and the Neither SU nor SGLT2i (active-treated comparator) group.

| Model | Patients | Events | Incidence density  (per 1000 person-year) | Effect estimate (95%CI) | p |
| --- | --- | --- | --- | --- | --- |
| Crude incidence rate  SU  Neither SU nor SGLT2i | 1448  3054 | 189  421 | 42.67  42.71 | 0.999 (0.842-1.186)  Ref | 0.993 |
| Unadjusted Cox model (HR)  SU  Neither SU nor SGLT2i | 1448  3054 | 189  421 | -  - | 0.908 (0.765-1.078)  Ref | 0.270 |
| Adjusted Cox model (HR)  SU  Neither SU nor SGLT2i | 1448  3054 | 189  421 | -  - | 0.870 (0.732-1.033)  Ref | 0.112 |
| Multivariable logistic regression (adjusted OR)  SU  Neither SU nor SGLT2i | 1448  3054 | 189  421 | -  - | 0.893 (0.740-1.075)  Ref | 0.235 |

Incidence density was calculated as the number of events divided by total person-years, multiplied by 1,000. For crude incidence, the effect estimate is the incidence rate ratio (IRR). For Cox and logistic models, effect estimates are hazard ratios (HRs) and odds ratios (ORs), respectively. The adjusted Cox and adjusted logistic models were adjusted for age, sex, aCCI, and baseline biguanides use. Neither SU nor SGLT2i was treated as the reference group. Abbreviations: SU, sulfonylureas; SGLT2i, sodium-glucose cotransporter-2 inhibitors; CI, confidence interval.


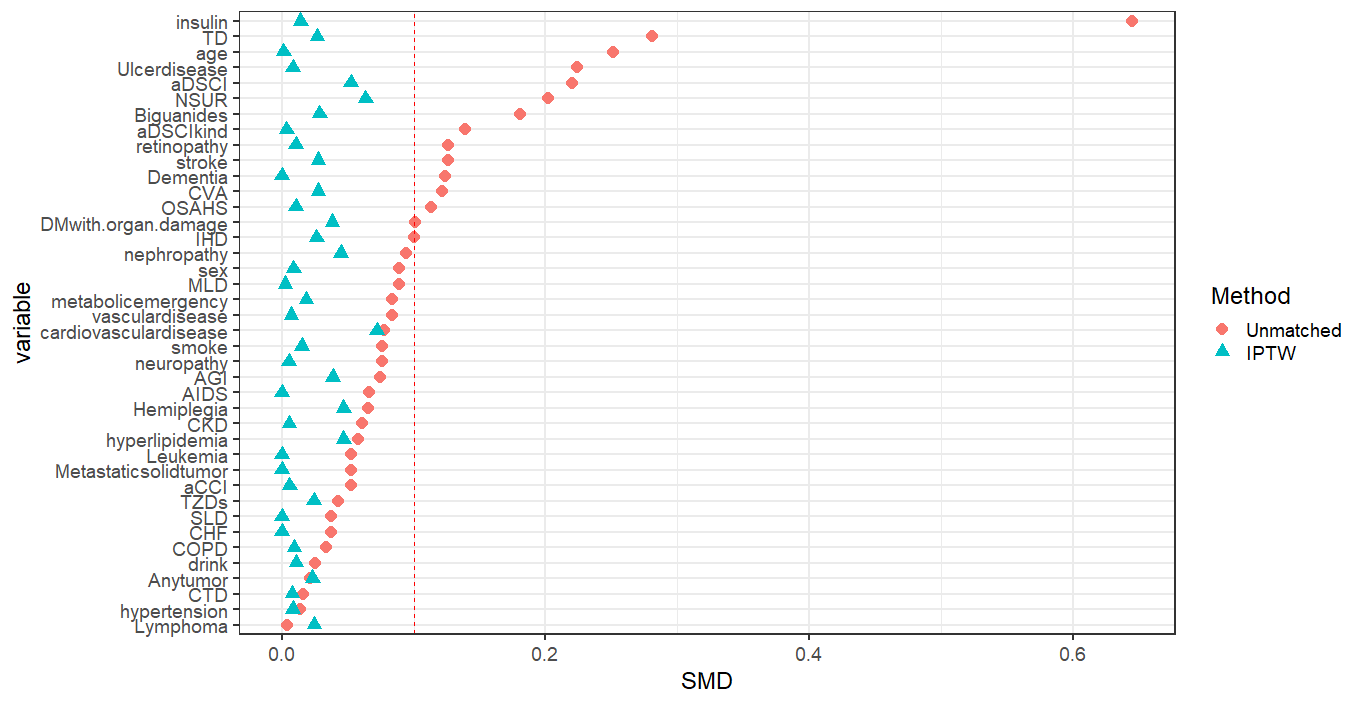


**Figure S1. The distribution of variables after IPTW.**

Abbreviations: TD, thyroid disease; aDCSI, the adapted diabetes complications and severity index; NSUR, non-sulfonylureas; CVA, cerebral vascular disease; OSAHS, obstructive sleep apnea-hypopnea syndrome; IHD, ischemic heart disease; MLD, mild liver disease; AGI, alpha-glycosidase inhibitors; AIDS, acquired immune deficiency syndrome. CKD, chronic kidney disease; aCCI, the age-adjusted charlson comorbidity index; TZDs: thiazolidinediones; SLD, moderate or severe liver disease; CHF, congestive heart failure; COPD, chronic pulmonary disease; CTD, connective tissue disease; IPTW, inverse probability of treatment weighting; SMD, standardized mean differences. SMD>0.1 indicates a non-negligible difference between the two treatment groups.
